# Brain Networks Supporting Execution of Mathematical Skills versus Acquisition of New Mathematical Competence

**DOI:** 10.1371/journal.pone.0050154

**Published:** 2012-12-10

**Authors:** Samuel Wintermute, Shawn Betts, Jennifer L. Ferris, Jon M. Fincham, John R. Anderson

**Affiliations:** Department of Psychology, Carnegie Mellon University,Pittsburgh, Pennsylvania, United States of America; Universidad de Granada, Spain

## Abstract

This fMRI study examines how students extend their mathematical competence. Students solved a set of algebra-like problems. These problems included Regular Problems that have a known solution technique and Exception Problems that but did not have a known technique. Two distinct networks of activity were uncovered. There was a Cognitive Network that was mainly active during the solution of problems and showed little difference between Regular Problems and Exception Problems. There was also a Metacognitive Network that was more engaged during a reflection period after the solution and was much more engaged for Exception Problems than Regular Problems. The Cognitive Network overlaps with prefrontal and parietal regions identified in the ACT-R theory of algebra problem solving and regions identified in the triple-code theory as involved in basic mathematical cognition. The Metacognitive Network included angular gyrus, middle temporal gyrus, and anterior prefrontal regions. This network is mainly engaged by the need to modify the solution procedure and not by the difficulty of the problem. Only the Metacognitive Network decreased with practice on the Exception Problems. Activity in the Cognitive Network during the solution of an Exception Problem predicted both success on that problem and future mastery. Activity in the angular gyrus and middle temporal gyrus during feedback on errors predicted future mastery.

## Introduction

Considerable research has examined the neural basis of mathematical competence (e.g., [1]–[6]). The **triple-code** theory (e.g., [7]) proposes that the horizontal intraparietal sulcus **(HIPS)** processes numerical quantities, a left perisylvian language network is involved in the verbal processing of numbers, and a ventral occipital-parietal region processes visual representations of digits. In related work, Dehaene et al. [8] identified three distinct parietal regions of interest: the HIPS quantity processing region, the angular gyrus **(ANG)** that is part of the perisylvian language network, and the posterior superior parietal lobule (**PSPL**, not part of the original triple-code theory) that supports attentional orientation on the mental number line and other spatial processing.

The ACT-R [9]–[10] models of equation solving [11]–[12] and mental multiplication [13] emphasize the contribution of two regions. One is the lateral inferior prefrontal cortex **(LIPFC)** associated with retrieval of declarative facts, including arithmetic facts and facts about algebra [14]. It is particularly involved in more advanced tasks involving topics like algebra, geometry, or calculus (e.g., [15]–[17]). The other region is the posterior parietal cortex **(PPC)**, which is about 2 centimeters away from each of the three parietal regions (HIPS, PSPL, and ANG) identified by Dehaene et al. [8]. The ACT-R theory associates this region with operations on mental representation of things like equations.

**Figure 1:**
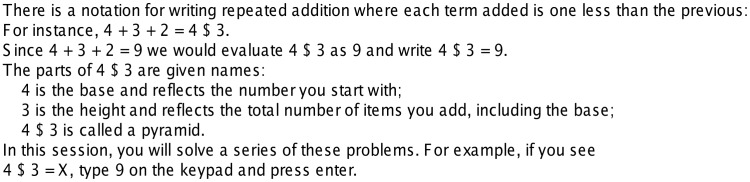
Instructions for participants.

Most of the prior research has focused on problems where students have mastered and practiced algorithms for solving the problems. In an attempt to go beyond such routine tasks, Anderson et al. [18] introduced the use of **pyramid problems** (Figure 1 gives instruction on pyramid problems used in our experiment). An example of a pyramid problem is 9$3 = X which is solved as 9+8+7 =  24. Pyramid problems involve a base (“9” in this example) that is the first term in an additive sequence and a height (“3” in this example) that determines the number of terms to be added. Each new term added in the sequence is one less than the previous term. As students work with pyramid problems they quickly master the algorithm for them. Nonetheless, students can still be placed in situations that require they extend their knowledge, which students can do so with at least some success. For instance, they can be presented with problems like these:

-5$4 = X

5$-4 = X

105$4 = X

5½$4 = X

X$4 = X.

The Anderson et al. study contrasted **Exception Problems** like the above with **Regular Problems** (defined as involving single positive digits for bases and heights). The contrast between these types of problems identified two patterns of activity that are localized in different brain regions. The first pattern, which we call “cognitive”, involves equal activity for Exception and Regular Problems. Additionally, the activity was much greater when solving the problem than when reflecting on the problem's solution, which was presented after students produced an answer. The second pattern, which we call “metacognitive”, involves greater activity for Exception Problems than Regular Problems and showed no decrease during the reflection period. More than 20% of the brain showed a significant (p<.01) correlation with one or both of these factors.

The names of these patterns, “cognitive” and “metacognitive”, reflect basic assumptions about the processes involved in solving these problems: Regular problems require similar processes to those incorporated in many existing cognitive models, such as retrieval of memories and manipulation of representations. However, Exception problems are not as straightforward to model. Part of that difficulty is the lack of good models of metacognitive processing, which is likely necessary to solve Exceptions. A person solving an Exception problem for the first time needs to be aware of the fact that their existing strategy will not work, and of how their objective relates to what they know how to do. For convenience in this study, we use the term “metacognitive” more broadly to indicate that set of processes which is used to understand and solve exception problems. This likely includes the above processes, but also some that are not usually called “metacognition”, such as drawing analogies.

**Figure 2:**
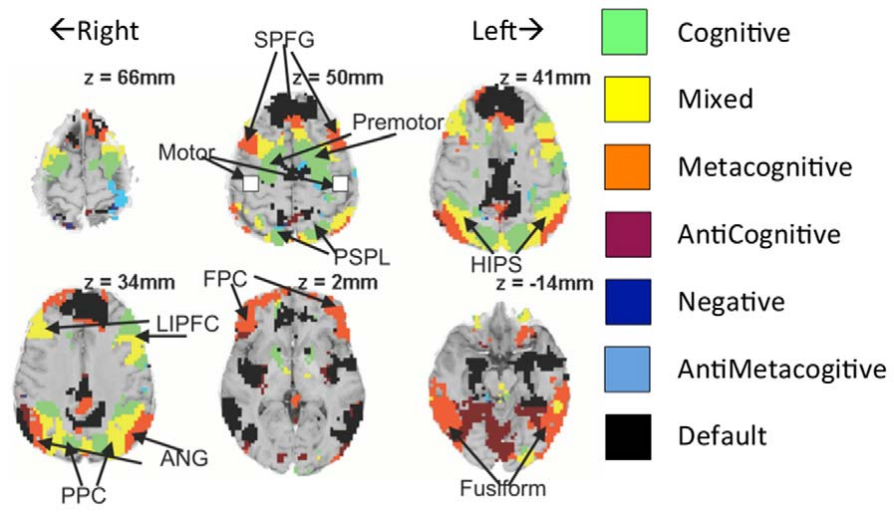
Classification of different brain regions in terms of their activation patterns (from [**18**]): Cognitive regions respond equivalently to Regular and Exception Problems and respond more strongly during problem solution than during reflection on feedback. Metacognitive regions respond more strongly to Exception Problems than Regular Problems and respond as strongly during problem solution and as reflection. Mixed regions also respond strongly to the task, but show an average of the cognitive and metacognitive patterns. See [18] or later in the text for an explanation of other regions.

Figure 2 shows the different categories of engagement and their distribution across the brain in this prior study, revealing what we have called the **Cognitive** and **Metacognitive Networks**. The prefrontal cortex displays a classic posterior-to-anterior gradient in abstraction (e.g., [19]–[22]), going from cognitive to metacognitive areas. In addition, the posterior cortex displays a medial-to-lateral gradient. The PSPL and the PPC regions show strong cognitive patterns; the HIPS and the LIPFC show a more mixed pattern; while the. ANG shows a strongly metacognitive pattern. In addition, there are strong cognitive patterns in the premotor region and metacognitive patterns in superior prefrontal gyrus, frontopolar regions, and the fusiform area. Note that all of these patterns are bilateral. The mixed and metacognitive regions show a substantial overlap with the flexibility areas in the recent meta-analysis of executive function by Niendam et al. [23].

### The Current Study

In the work of various labs, there is an emerging understanding of routine mathematical behavior. For instance, there is an ACT-R model of what is happening in the Cognitive Network during the solution of Regular Problems (available at the website associated with this paper: http://act-r.psy.cmu.edu/publications/pubinfo.php?id=1020). The goal of the current study is to understand what is happening in the Metacognitive Network and how both networks support learning to solve Exception Problems. More generally, the goal of this research is to understand how students extend their mathematical competence. The Anderson et al. study [18] was limited by the infrequent appearance of Exception Problems. Thus, there were not enough observations to determine which aspects of Exception Problems were driving the patterns of activation. The current study contrasts two kinds of Exception Problems: Some (like -5$4 = X) involve unusual arguments like negative numbers, and large numbers, but are solved by the same additive procedure. Others (e.g., X$4 = X) require a change to the solution algorithm (guess-and-check is the most common method for this type of problem). We will refer to these two types of problems as involving **Argument Exceptions** versus **Algorithm Exceptions**.

Also, because of the infrequent use of Exception Problems, the previous study could not address the role of the Cognitive and Metacognitive Networks in learning. Any particular Exception Problem type only appeared once and so gave no opportunity to investigate how mastery of that type would develop and what the accompanying activity changes would be. In the current experiment participants solved 8 examples of each exception type, enabling us to track learning.

In the first day of this experiment participants received the instruction on Regular Problems (see Figure 1) and practiced solving such problems (i.e., solving for X in 9$3 = X). On the second day Exception Problems were introduced. The nine types of Exception Problems are shown in Table 1, classified by whether they are Argument, Algorithm, or Dual Exceptions. Argument Exceptions just involve repeated addition but one of the arguments is unusual. Algorithm Exceptions just involve single digit base and height but require a change to the algorithm. Dual Exceptions involve both unusual arguments and a change to the algorithm. Table 1 also orders the problems within each of these categories as to their anticipated difficulty and shows the feedback explaining the solution for each problem type.

**Table 1:** The nine types of exception problems used and mean accuracy and latency for correct answers, as well as number of correct trials contributing to the analysis of the fMRI data.

	Easy	Medium	Hard	Combined
Exception Category	Argument	Argument	Argument	Argument
Type	Negative Height	Negative Base	Large Base Unknown Value	
**Example**	4$-3 = X	-2$4 = X	208$3 = X	
**Accuracy**	84.40%	72.60%	63.50%	73.50%
**Latency (sec.)**	11.48	15.06	17.54	14.7
**n**	244	207	184	633
**Exception Category**	Algorithm	Algorithm	Algorithm	Algorithm
**Type**	Unknown Height	Unknown Base	Double X	
**Example**	5$X = 12	X$4 = 30	X$X = 15	
**Accuracy**	85.40%	85.40%	66.00%	77.89%
**Latency (sec.)**	9.78	9.78	13.91	11.69 sec.
**n**	245	237	189	671
**Exception Category**	Dual	Dual	Dual	Dual
**Type**	Large Base Unknown Height	Fractional Height	Mirror	
**Example**	110$X = 434	5$2⅓ = X	200$401 = X	
**Accuracy**	89.90%	74.70%	62.20%	75.58%
**Latency (sec.)**	9.48	13.94	12.63	12.02
**n**	257	214	179	650
**All Types**				
**Accuracy**	86.60%	76.50%	63.90%	
**Latency (sec.)**	10.25	13.46	14.69	
**n**	746	658	552	

Example feedback given for each problem:

**4$-3 = X**: 4$-3 = 4+5+6 = 15 (or 5+6+7 if participant had chosen that solution).

**−2$4 = X**: −2$4 = −2+−3+−4+−5 = −14.

**208$3 = X:** 208$3 = 208+207+206 = 621.

**5$X = 12**: 5$3 = 5+4+3 = 12.

**X$4 = 30**: 9$4 = 9+8+7+6 = 30.

**X$X = 15:** 5$5 = 5+4+3+2+1 = 15.

**110$X = 434:** 110$4 = 110+109+108+107 = 434.

**5$2⅓ = X:** 5$2⅓ = 5+4+⅓(3) = 10.

**200$401 = X:** 200$401 = 200+199+…−199+−200 = 0.

**Figure 3:**
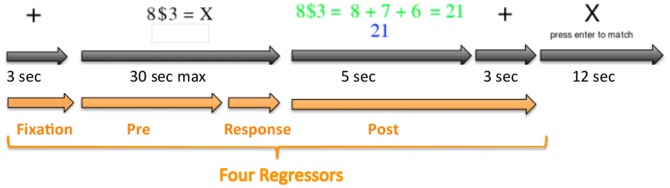
An illustration of the sequence of events for each problem: The problem began with a 3-second fixation and then was followed by a problem that stayed on the screen until the participant answered or until 30 seconds were up. Participants responded by entering the answer in a numerical keypad. This was followed by feedback on the correct answer and its expansion. After seeing the feedback for 5 seconds, there was another 3 seconds of fixation. After this, participants were given a repetition-detection task for 12 seconds. In this task letters appeared on the screen at the rate of 1 per 1.25 seconds. Participants were instructed to click a key each time they detected a pair of letters that were the same. Also illustrated are the four regressors used for analysis – a 3 second “Fixation” regressor corresponding to the initial fixation period, a “Pre” regressor for the period from problem presentation to response initiation, a “Response” regressor for the period of response generation, and a ‘Post” regressor for the 8 seconds until the start of the repetition detection.

As illustrated in Figure 3, subjects were given 5 seconds to study feedback on their solution. We obtained separate estimates of activity for the period before the response was entered (**Pre** response) and the period during the feedback after response (**Post** response). We expected Cognitive and Metacognitive regions would be distinguished by their differential activity in the Pre period and the Post period and by their differential activity to regular problems versus exception problems, following two broad patterns:

#### Cognitive Pattern

Cognitive regions should be principally engaged during the solution of the problem and not during reflection on feedback. There should be no difference between Exception Problems and Regular Problems.

#### Metacognitive Pattern

There should be no difference in the engagement of metacognitive regions before or after the response, as these regions should be engaged both in problem solution and reflection on feedback. However, they should be more engaged when dealing with an Exception Problem.

As in Anderson et al. [18] we created a contrast between activity during regular problems in the Pre period (PreReg) and activity during exception problems in the Post period (PostEx) to serve as the primary differentiation between Cognitive and Metacognitive regions. The expectation is that Metacognitive regions should show more activity during PostEx than during PreReg, indicating that the region is more involved with reflecting on exceptions than with routine mathematical cognition. In contrast, Cognitive regions should show greater PreReg than PostEx activity, indicating that the region is more active during routine cognition than while reflecting on exceptions.

## Methods

### Participants

Fifty right-handed participants were recruited for the behavioral portion of the experiment (21 female, 29 male, ages 18–40, mean = 23.4). Of those 50, 39 continued to the fMRI portion of the experiment (16 female, 23 male, ages 19–35, mean = 23.05). Ten did not participate in the fMRI portion due to poor performance (see below), and one participant did not show up.

### Procedure: Behavioral Training Session

The first session, outside of the scanner, involved 81 Regular problems, where the base was in the range 1–9 and the height 1–9. Each problem was presented on the screen as shown in Figure 3 preceded by a 3 second fixation period. Participants had 30 seconds to input an answer using a numeric keypad. After their response or 30 seconds expired, feedback was presented for 5 seconds, showing the explanation for the correct answer (see Table 1) and indicating whether their response was correct. After feedback, a fixation cross was again presented for 3 seconds, followed by a simple repetition detection task for 12 seconds. During repetition detection, letters appeared on the screen at a rate of 1 per 1.25 seconds, and participants were instructed to press enter on the keypad when repeated letters occurred. This task served to distract the participants from the main pyramid task and return brain activity to a relatively constant level.

On the first day, prior to solving the problems, participants were trained to enter values on a numeric keypad without looking. While solving problems, the keyboard was concealed from the participants' view, mimicking conditions in the scanner. Participants who solved less than 32 of the last 45 problems correctly (due to calculation error or inability to use the concealed keypad) were not allowed to continue to the fMRI session.

### Procedure: fMRI Session

The scanner session occurred within two days of the behavioral session. Problems were divided into nine blocks, and were presented the same way as in training, with the same timing. The first block, during structural image acquisition, consisted of 16 Regular Problems (base ranging from 4–9 and height from 2–5). The remaining 8 blocks, which provide the data for the experiment, each consisted of one Regular Problem to start, which was not analyzed, followed by a randomly ordered mix of 9 Exception and 2 Regular Problems. Each of these blocks had exactly one problem of each exception type. Due to time limitations, one participant did not participate in the last block and one participant did not participate in the last two blocks. Data from the earlier blocks for these participants were analyzed, however. Based on preliminary analysis of the scan data, three participants with abnormally large changes in BOLD signal were detected. These participants were excluded from further analysis, leaving 36 participants.

Images were acquired using gradient echo-echo planar image (EPI) acquisition on a Siemens 3T Verio Scanner using a 32 channel RF head coil, with 2 s. repetition time (TR), 30 ms. echo time (TE), 79° flip angle, and 20 cm. field of view (FOV). The experiment acquired 34 axial slices on each TR using a 3.2 mm thick, 64×64 matrix. This produces voxels that are 3.2 mm high and 3.125×3.125 mm^2^. The anterior commissure-posterior commissure (AC-PC) line was on the 11^th^ slice from the bottom scan slice. Acquired images were pre-processed and analyzed using the NIS system and AFNI [24]–[25]. Functional images were motion-corrected using 6-parameter 3D registration (AIR; [26]). All images were then co-registered to a common reference structural MRI by means of a 12-parameter 3D registration and smoothed with an 6 mm full-width-half-maximum 3D Gaussian filter to accommodate individual differences in anatomy.

### fMRI Analysis

As in Anderson et al. [18], the critical analyses required estimates of the activity before and after the response (i.e. Pre and Post activity). To separate these two estimates from any activity that involved preparation for the upcoming trial or response generation, we used four regressors – one for the initial 3-second fixation period, a Pre regressor for the variable period up to the first keypress of the response, one from that time to response completion, and a Post regressor for the 8 seconds after response completion. These regressors were created by taking boxcar functions for the four periods and convolving them with a hemodynamic response function. We used the same hemodynamic function as used in Anderson et al. ([18] – a gamma function with an index parameter of six and a scale parameter of 0.75 seconds).

We obtained separate estimates of Pre and Post activity for the Regular Problems, Argument Exceptions, Algorithm Exceptions, and Dual Exceptions. In addition, although most of the analyses will be on correct problems, we obtained separate estimates for problems solved correctly and incorrectly. Finally, there were separate regressors to deal with the cases where participants timed out and never generated a response. Thus, there are a potential of 36 possible problem-related regressors (4 periods×4 types×2 correctness possibilities, plus 4 timeout regressors). However, the fixation values were constrained to be the same for all conditions, as were the response values, resulting in 20 regressors. Finally, a set of regressors for a quadratic function was added to the analysis for each block to extract any general trends. Using these regressors allowed us to estimate measures of activity in different critical time periods. We will refer to these as **estimates of engagement**.

These estimates of engagement we obtained for different predefined and exploratory regions. For predefined regions, we focused on the HIPS, PSPL, and ANG from Dehaene et al. [8], and the LIPFC and PPC from the ACT-R theory [7]. These regions have been previously used by Rosenberg-Lee et al. [13] and Anderson et al. [18]. As in [18], the coordinates for the HIPS region were updated to reflect the larger meta-analysis of Cohen et al. [27]. Left and right analogs were used for each region. The locations of the regions are:

- **PSPL:** a 12.8 mm. (high) by 12.5×12.5 mm^2^ region centered at Talairach coordinates +/−19,−68,55 in Brodmann Area 7.
- **HIPS:** a 12.8 mm. (high) by 12.5×12.5 mm^2^ region centered at Talairach coordinates +/−34,−49,45 in Brodmann Area 40.
- **ANG:** a 12.8 mm. (high) by 12.5×12.5 mm^2^ region centered at Talairach coordinates +/−41,−65,37 in Brodmann Area 39.
- **LIPFC:** a 12.8 mm. (high) by 15.6×15.6 mm^2^ region centered at Talairach coordinates +/−43,23,24 spanning Brodmann Areas 9 and 46.
- **PPC:** a 12.8 mm. (high) by 15.6×15.6 mm^2^ region centered at Talairach coordinates +/−23,−63,40 spanning Brodmann Areas 7 and 39.

An exploratory analysis was performed using the Pre-Regular versus Post-Exception contrast to find cognitive and metacognitive regions.. This exploratory analysis looked for regions of at least 10 contiguous voxels that showed a voxel-wise significance of 0.00003 for the difference between Pre-Exception and Post-Regular. Using these values results in a brain-wide significance estimated to be less than 0.01 by simulation [24], [25].

## Results

Results reported here will focus on the last 8 blocks of the fMRI session where functional imaging data are available. A major goal of this study was to understand how students learned to master exception problems. To examine learning trends we broke data down into four quarters (2 blocks each).

### Behavioral Results

**Figure 4:**
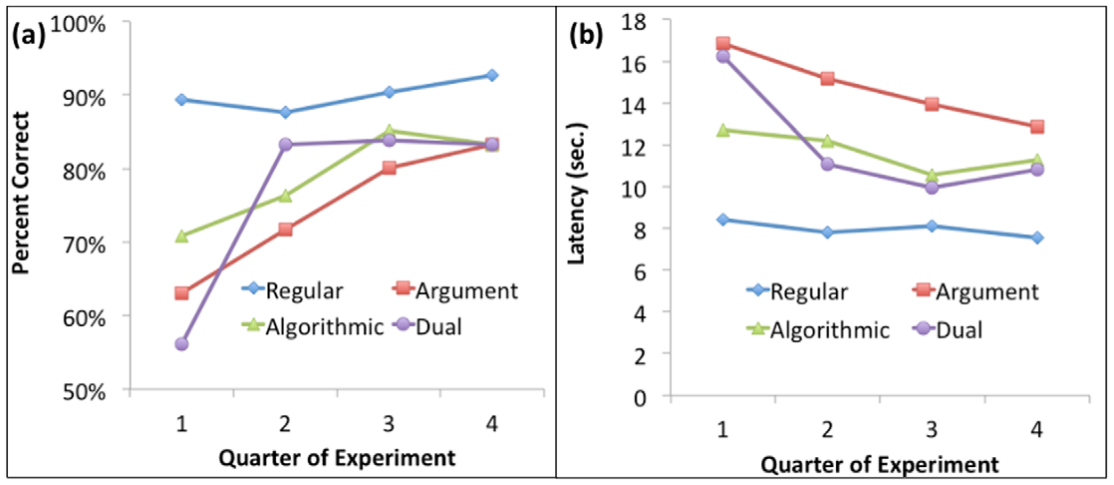
Behavioral results in scanner session as a function of type of problem and quarter of the experiment: (a) Percent Correct; (b) Mean response time for correct responses.

Figure 4 shows the accuracy and latency broken down according to type of problem and quarter (average of two blocks) of the experiment. The latency is for correct responses only. In an analysis that contrasts Regular Problems versus the average of all Exceptions, there are significant effects of quarter (accuracy: F(3,105) = 17.10, p<.0001; latency: F(3,105) = 22.18, p<.0001), type of problem (accuracy: F(1,35) = 54.69, p<.0001; latency: F(3,105) = 226.29, p<.0001), and an interaction between the two (accuracy: F(3,105) = 11.58, p<.0001; latency: F(3,105) = 17.00, p<.0001) such that the differences among conditions decreases with practice. A contrast of the difference between Exceptions and Regulars in the first quarter and the average difference in the last two quarters is highly significant (accuracy: F(1,105) = 32.74, p<.0001; latency: F(1,105) = 48.40, p<.0001) and the remaining variance in the interaction is not significant (accuracy: F(2,105) = 1.00, p>.25; latency: F(2,105) = 1.29, p>.25)

We also did separate analysis of the Exception Problems, classifying them by type and difficulty. Table 1 presents the mean accuracy and latency for the 3×3 exception types. There are again significant effects of quarter (accuracy: F(3,105) = 33.54, p<.0001; latency: F(3,105) = 39.92, p<.0001). With respect to difficulty, there is a significant main effect (accuracy: F(2,70) = 50.84, p<.0001; latency: F(2,70) = 85.16, p<.0001) and an interaction with quarter (accuracy: F(6,210) = 2.57, p<.05; latency: F(6,210) = 2.42, p<.05) such that the differences among difficulty levels decreases with practice. The effects of type are of more interest: no significant effect of type on accuracy but an effect on latency (accuracy: F(2,70) = 1.35, p>.25; latency: F(2,70) = 35.62, p<.0001) and a highly significant interaction between type and quarter (accuracy: F(6,210) = 4.29, p<.0005; latency: F(6,210) = 8.48, p<.0001). This interaction is largely driven by the large improvement of the Dual problems from the first quarter to later quarters. The three Dual Exceptions all involve learning algorithms that avoid adding large numbers or dealing with fractions. In contrast, participants have to continue to add the difficult numbers for the Argument problems, and so latency (and to some degree accuracy) remains worse for these throughout the experiment. Only latency shows a significant interaction between difficulty and type (accuracy: F(4,140) = 1.76, p>.10; latency: F(4,140) = 8.87, p<.0001). The three-way interactions are not significant for either measure.

The accuracy differences among the exception types have disappeared by the final quarter (the three means are actually identical) and latency differences have been reduced. The differences between Regular and Exception Problems have been reduced as well. This suggests that participants are reaching some level of mastery of these Exception Problems. Furthermore, there is little change in the last half of the experiment (there is no significant effect for either measure of quarter or interaction with quarter for the last two quarters), suggesting an end to an initial phase of learning.

### Imaging analysis: Predefined regions

Most of the imaging analyses used data from correctly answered problems only. Table 1 gives the number of correct cases for each exception type. The number of correct regular cases is 512. As described in the Methods section we extracted measures of engagement before and after the period of response generation. To deal with issues of non-sphericity in our main statistical tests of regional activity, we reduced all factors to binary contrasts. For the effect of practice, we contrasted the first quarter with the average of the last two quarters since accuracy and latency asymptote over the last two quarters. We collapsed the exception types in the analyses of variance, although we will report binary tests between the different types as well. Table 2 reports the main effects of an analysis of variance as signed *t*-tests (square root of *F* statistic) so that the direction of the effect is apparent. A positive *t* indicates left hemisphere greater than right, Pre-response activity greater than Post-response activity, activity in the first quarter greater than later, and activity for Exceptions greater than Regulars. The table reports *t*'s for the two interactions of interest. Both look at how other factors modulate the effect of problem type: whether the Exception-Regular difference is greater before than after the response, and whether it is larger earlier than later in the experiment (while there were some interactions with hemisphere, in all cases this took the form of the effect being stronger in the dominant hemisphere but not changing its direction). The table also reports tests for differences among the four types of problems and a test for whether the activity after the response for Exceptions was greater than the activity before the response for Regulars. A positive value on this last test (PostEx-PreReg) indicates a metacognitive region and a negative value indicates a cognitive region. Finally, the table reports tests of whether the Pre and Post activity is greater for Exception Problems incorrectly solved than Exception Problems correctly solved. There were not enough observations of errors to allow a similar contrast for Regular Problems.

**Table 2:** Predefined Regions: Critical Statistical tests reported as *t*-values.

		PPC	LIPFC	PSPL	HIPS	ANG
Main Effects	**Left>Right**	0.68	1.15	−0.87	5.27***	4.61**
	**Pre>Post**	5.67***	6.73***	3.69*	4.69**	−3.60*
	**1>(3,4)**	0.74	1.28	1.04	−0.12	0
	**Ex>Reg**	6.07***	6.88***	4.12**	6.97***	4.21**
Type Difference Effects	**Pre>Post**	0.02	3.73*	0.02	2.21	3.43
	**1>(3,4)**	0.6	−0.18	0.51	0.65	2.4
Exception Type Contrast	**Arg - Reg**	2.29	3.75*	2.14	3.26	1.62
	**Alg - Arg**	5.32***	4.74**	2.75	5.70***	4.41**
	**Dual - Alg**	3.13	0.99	2.55*	0.14	0.44
Metacognitive Contrast	**PostEx-PreReg**	−3.1	−2.74	−1.57	−1.18	4.91**
Error-Correct (Exceptions)	**Pre**	−2.08	−0.91	−2	−0.79	0.23
	**Post**	7.07***	5.96***	4.60**	10.98***	3.50*

Significance levels for a 2-tailed *t*-test with 35 degrees of freedom, correcting for multiple tests by the Holm-Bonferroni method:

* *p*<.05.

** *p*<.01.

*** *p*<.001.

The PPC, LIPFC, PSPL, and HIPS show similar effects. There is greater activity before the response to Regulars than after the response to Exceptions. None of the t-tests for these effects reach significance, although the effects for PSPL and HIPS would if the conservative Holm-Bonferroni correction were not used (all four contrasts were significant in [18]). The pattern for ANG is quite different, showing greater response in the Post period and therefore showing a positive Post-Exception versus Pre-Regular contrast, indicative of a metacognitive region.

Among the exception types, Argument Exceptions evoke the least response and Dual Exceptions the greatest. All regions show this same ordering, although the Dual-Algorithmic difference is not significant for LIPFC, HIPS, or ANG. All regions show less engagement (and at high levels of significance) for Argument Exceptions even though these problems are the most difficult in terms of latency.

**Figure 5:**
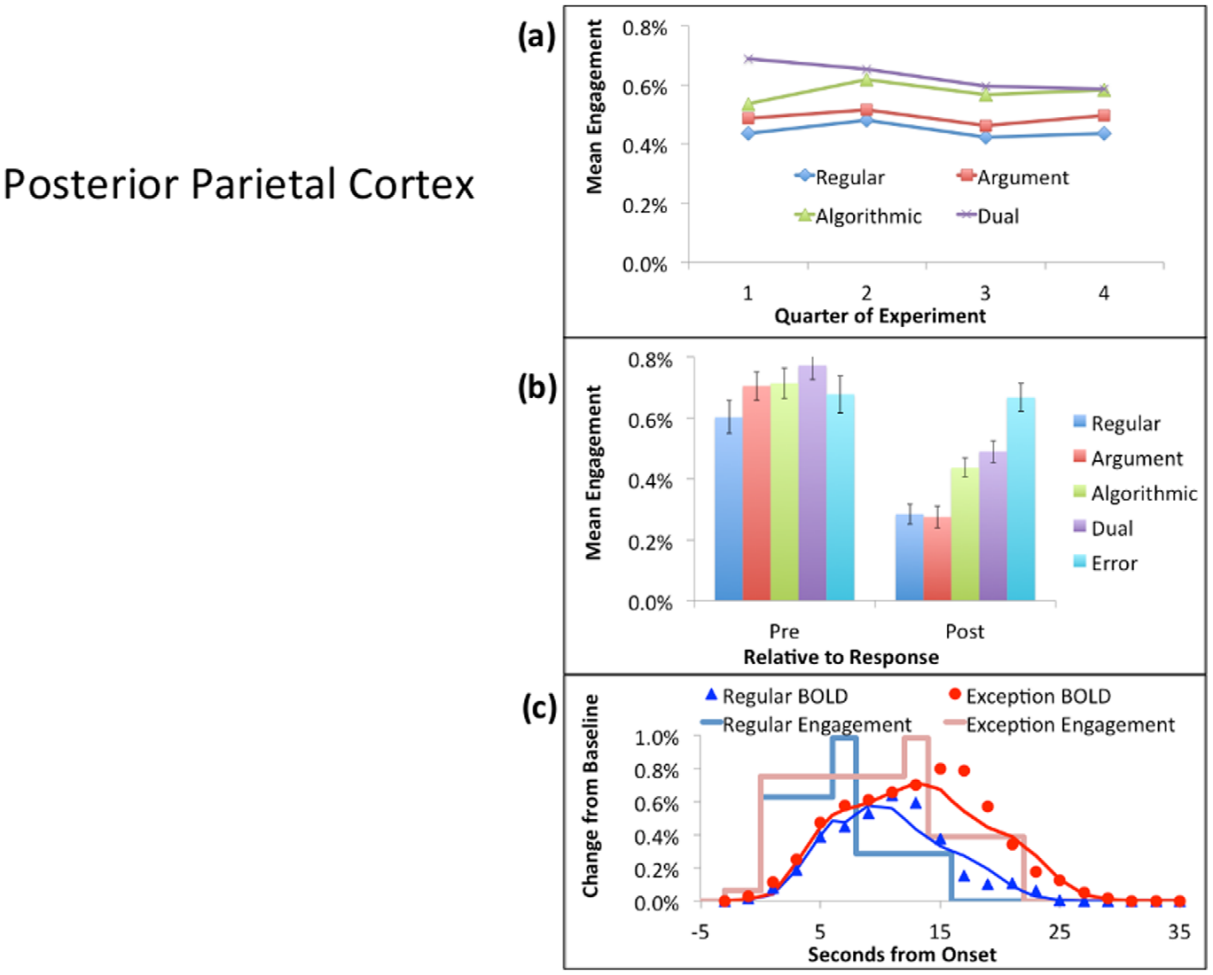
Activity in the PPC: (a) The mean of the Pre and Post Activity over the four quarters of the experiment for correctly solved instances of the different problem types. (b) The Pre versus Post activity for correctly solved instances of the four problems types plus activity for Exception errors. (c) The hemodynamic response for correctly solved Regulars and Exceptions (averaged over types) using the event locking procedure described in [13]. The boxcar lines give the average engagement estimated for the Fixation, Pre, Response, and Post periods. The points are the data and the solid line is the predicted BOLD response obtained by convolving a hemodynamic response function with the engagement functions.

Since the PPC shows the most negative PostEx-PreReg contrast, Figure 5 displays it as the most cognitive pattern. As can be seen in part (a) of the figure, there is little change in its response over the course of the experiment. Participants are speeding up for Exception Problems (an average of about 4 seconds), but the engagement per unit time is not changing. Figure 5b shows that, while there is some variation in the engagement to different types of correctly solved problems, the larger effect is the 50% decrease from the Pre period to the Post period.

**Figure 6:**
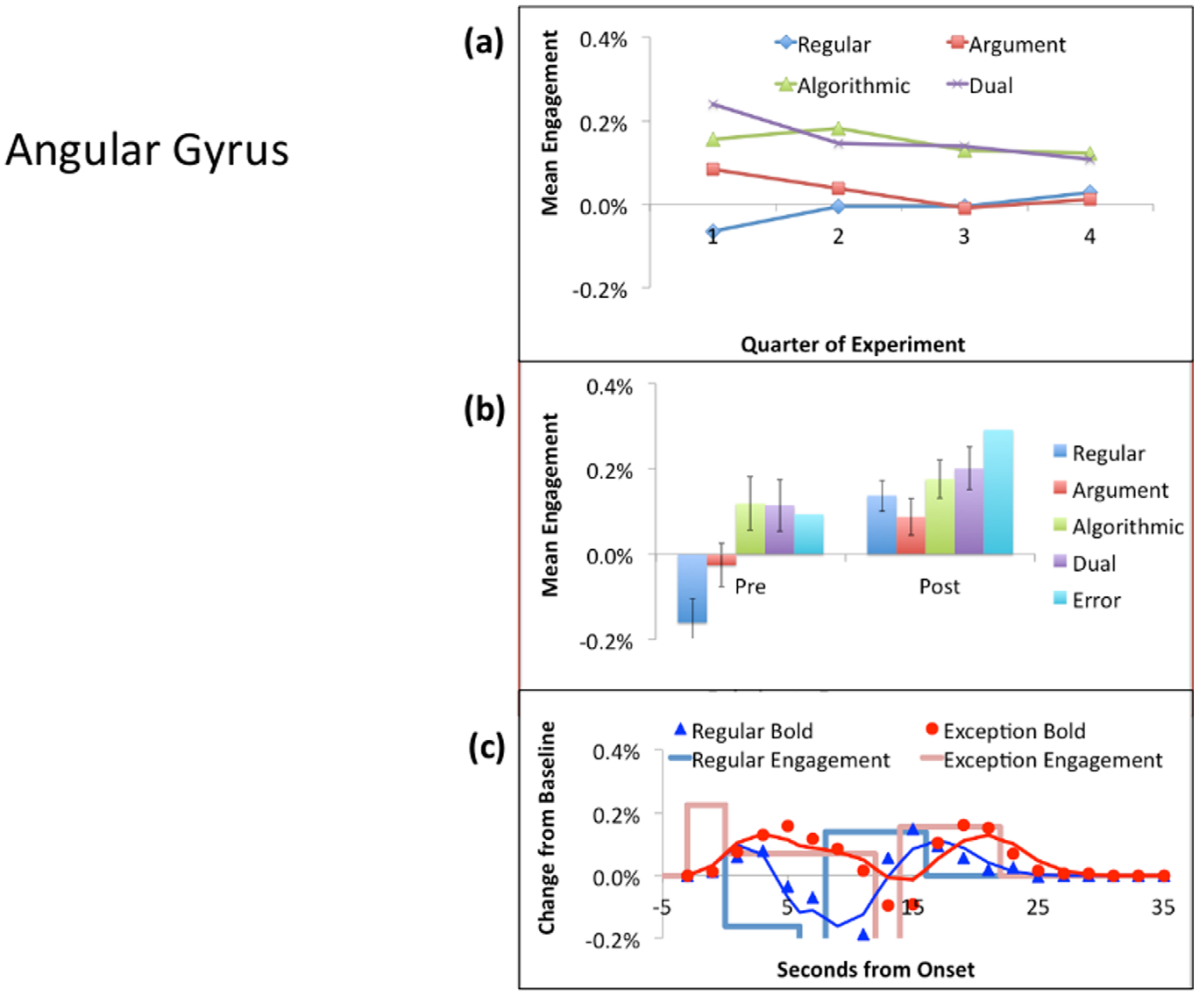
Activity in the ANG: See **Figure 5** for a description of the figure. The mean estimated engagement during the response period (not shown in part c) drops to −.50%,

Figure 6 shows the results for the ANG, which has the only positive PostEx-PreReg contrast and hence exemplifies the metacognitive pattern. Part (a) of the figure shows that the average response of this region for Regular Problems is close to zero. Exception Problems show a positive response that decreases over time. Figure 6b shows that the activity increases in the Post period. Regular problems switch from quite negative to quite positive, resulting in a fairly strong interaction between problem type and the Pre versus Post period. In Figure 6c, note the early rise in estimated engagement. This estimate is for the fixation period before the participant knows the type of problem they will encounter. This strong early engagement occurs both for Regulars and Exceptions. In contrast, once the response is determined and the participant is keying it out during the response period, there is a strong negative dip in the hemodynamic response. We have consistently found a negative response in this region during periods of routine behavior, including well-practiced mathematical tasks (e.g., [13]).

The results replicate and extend the results reported in Anderson et al. [18]. Of the 5 predefined regions, the ANG is again the only one to show the metacognitive pattern. Also, the ANG was the only region to show a significant reduction in the difference between regular and Exception Problems with practice. The region seems to be disengaging as the problems become more familiar.

The results for the different types of Exceptions indicate that it is a change in the algorithm that is mainly responsible for the increased response to Exception Problems, and not a change to more complex arguments. Even though Argument Exceptions were the most difficult in terms of latency, the Algorithmic and Dual Exceptions produced significantly greater activation in all predefined areas.

We also found that these regions respond more to errors, but only upon receiving feedback (the Post period). There was a tendency for cognitive regions to respond less while the error was being made. This effect was significant for the PPC. Errors also produced less Pre activity in the cognitive regions in [18], although the effects were not significant.

### Imaging analysis: Exploratory regions

**Figure 7:**
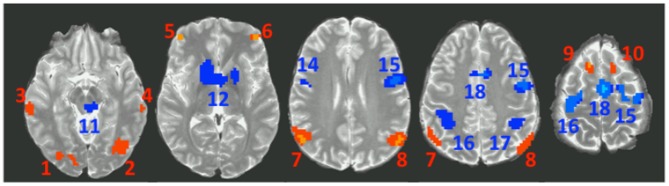
Exploratory regions showing a significant effect of the contrast between activity before the response for Regulars and after the response for Exceptions. Red regions show the metacognitive pattern of greater engagement reflecting on the solution to Exception Problems. Blue regions show the cognitive pattern of greater activity solving Regular Problems.

The exploratory analysis that used the contrast between Post Exception and Pre Regular (see Table 3 and Figure 7) uncovered 10 metacognitive regions and 8 cognitive regions. The 10 metacognitive regions in Table 3a break out in to 5 pairs of left and right homologs. This includes regions (7 & 8) that overlap with the predefined ANG. The cognitive areas include regions (14 & 15) that border the LIPFC and regions (16 & 17) that overlap HIPS.

**Table 3:** Results for Exploratory Areas: Five Contrasts reported as t-values.

	Region of Interest	Brodmann Area(s)	Coordinates (x,y,z)	Voxel Count	1. Type Diff. 1>(3,4)	2. Algorithm - Argument	3. Dual - Algorithm	4. Error - Correct (Pre)	5. Error - Correct (Post)
(a) PostEx>PreReg									
1	R Occip./Temp.	18/19/37	26,−73,−7	242	1.37	1.93	3.18	−1.5	−1.21
2	L Occip./Temp.	18/19/37	−33,−69,−8	247	1.13	2.74	7.36**	−2.29	0.73
3	R Mid Temp. Gyr.	21	61,−28,−4	24	2.09	3.60*	0.47	0.23	4.07*
4	L Mid Temp. Gyr.	21	−58,−29,−4	10	3.99*	5.76***	−1.62	−1.42	2.25
5	R Mid/Inf Front. Gyr.	10	44,44,0	10	−0.62	3.01	1.54	−0.7	4.64**
6	L Mid/Inf Front. Gyr.	10	−38,44,−3	14	2.04	4.22*	1.22	−0.3	2.86
7	R Angular Gyr.	40	52,−54,38	181	0.71	2.63	1.44	0.45	4.95**
8	L Angular Gyr.	40	−50,−57,38	201	2.84	4.91**	−0.71	0.7	1.66
9	R Sup Front. Gyr.	6	14,21,55	29	−0.1	2.04	0.76	0.68	2.69
10	L Sup Front. Gyr.	6	−13,22,55	21	−0.54	2.3	−0.27	−0.19	0.19
	Average Metacognitive			979	2.22	3.90*	2.84	−0.89	2.88
(b) PreReg>PostEx									
11	R/L Thalamus		−2,−26,1	61	2.54	1.08	−0.77	−0.02	3.95**
12	Caudate, Putamen		7,8,5	422	−0.89	−0.17	0.75	−2.97	0.47
13	L Insula	13	−28,19,7	12	0.19	5.15***	−2	−2.74	6.84***
14	R Precentral Gyr.	46	49,−2,26	14	−1.53	1.91	1.12	−2.41	3.92**
15	L Front.	Jun-46	−27,−2,46	467	−0.45	2.57	−1.03	−2	5.45***
16	Pre&Post Cent. Gyr.	1,2,4,6,40	36,−18,52	266	−1.82	0.17	0.17	−2.67	1
17	L Inf Parie. Lob.	40	−36,−39,42	67	−0.52	3.29	−1.07	−1.19	4.78**
18	Medial Frontal	6,32	−2,3,48	283	−0.79	0.42	−0.64	−2.02	4.40**
	Average Cognitive			1593	0.19	1.14	−0.4	−2.75	3.88*

Significance levels for a 2-tailed t-test with 35 degrees of freedom, correcting for multiple tests by the Holm-Bonferroni method:

* p<.05.

** p<.01.

*** p<.001.

Table 3 gives the locations of these regions and *t*'s for five contrasts of interest:

1. Whether the Exception-Regular contrast decreases over the course of the experiment.
2. The difference between Algorithmic Exceptions and Argument Exceptions.
3. The difference between Dual Exceptions and Algorithmic Exceptions.
4. The difference between Errors and Corrects for Exceptions in the Pre period.
5. The difference between Errors and Corrects for Exceptions in the Post period.

**Figure 8:**
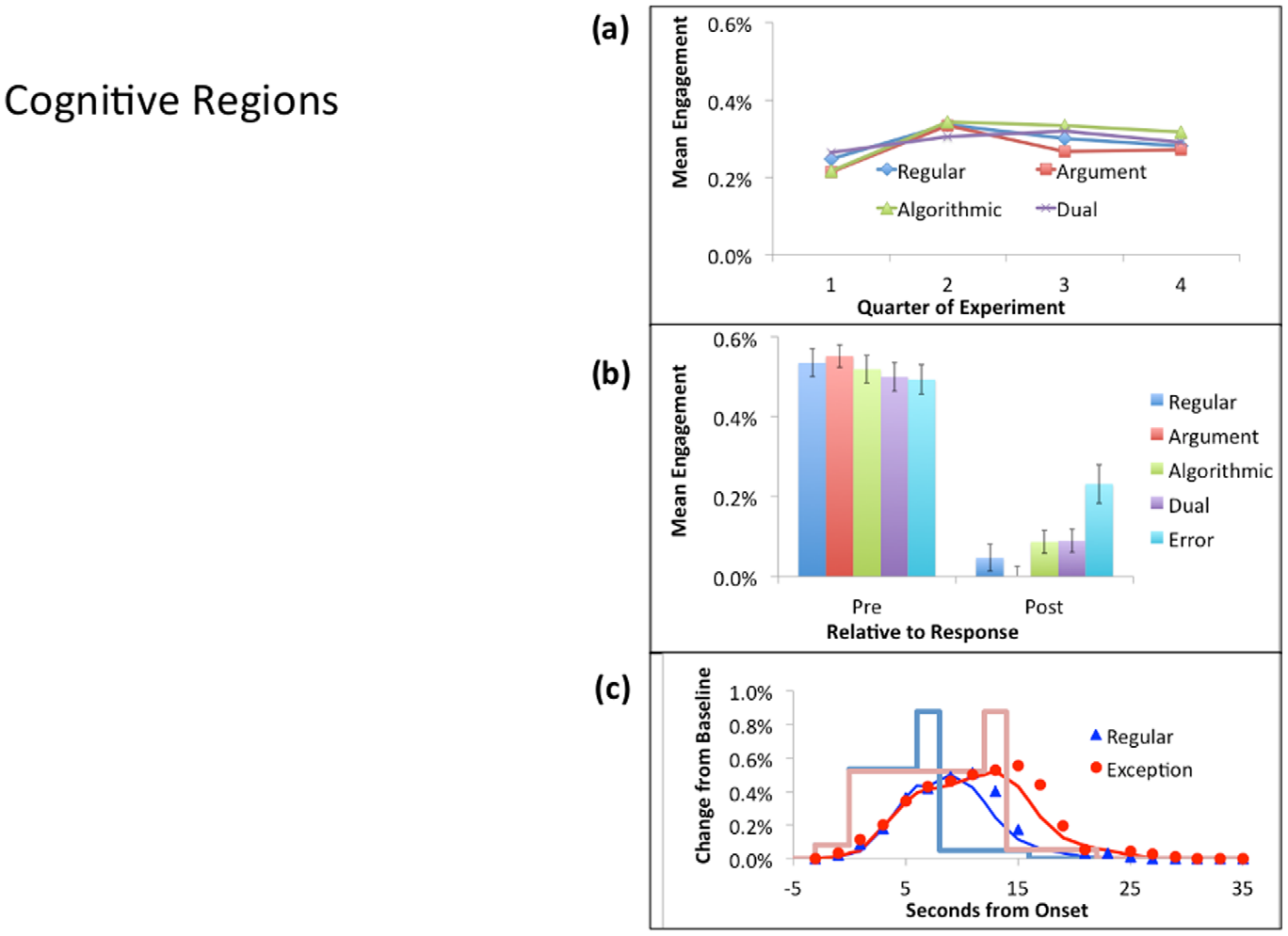
Average activity in the cognitive areas (**Table 3** & **Figure 6**). See **Figure 5** for a description of the figure.

**Figure 9:**
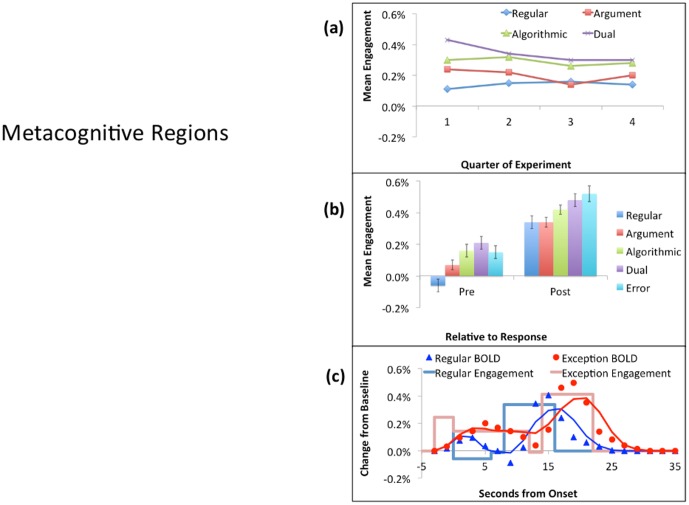
Average activity in the metacognitive areas (**Table 3** & **Figure 6**). See Figure 5 for a description of the figure.

Each of these contrasts is orthogonal to the Pre-Regular versus Post-Exception contrast that was used to select the regions. We combined the average response of the metacognitive regions, weighting their activity by the number of voxels in a region. We similarly averaged the 8 cognitive regions. Figures 8 and 9 display these average effects and Table 3 includes the 5 t-tests above for the average activity. The individual regions are largely consistent with the average effects. The differences among problem types (Contrasts 2–3) are significant only for the metacognitive average and not the cognitive average. There is less engagement on error trials before the response for the cognitive regions, but no such effect for the metacognitive regions (Contrast 4). The predefined cognitive regions showed a similar tendency. There is more activation after an error in both cognitive regions and metacognitive regions (Contrast 5), reflecting a rather unsurprising increased engagement to process the feedback.

For the cognitive areas in Figure 8, it is striking how little difference there is among the different correct problem types in the Pre-response period, and how little Post-response activation there is. These regions appear much “purer” cognitive regions than any of the predefined regions.

### Predicting Future Learning

Does engagement of the cognitive and metacognitive regions predict learning for the Exception Problems? To answer this question we used activity in these regions on the first block to predict performance on later blocks. The cognitive and metacognitive regions uncovered during the exploratory analysis were used. The dependent measure was the number correct solutions for an exception type on Blocks 2–8. As there are 9 exception types for each participant there 36*9 = 324 such measures of future learning. The five independent variables were amount of engagement before and after response for cognitive and metacognitive regions in Block 1 (2×2 = 4), plus a binary variable of whether the item was correct in that block. A stepwise multiple linear regression was performed on the 324 cases to determine which variables were significantly related to future performance. Table 4a reports the results of this analysis: There were significant positive contributions of being correct on Block 1, of cognitive activity before the Block 1 response, and of metacognitive activity after the Block 1 response.

**Table 4:** Results of the stepwise multiple regression analyses predicting number correct on Blocks 2–8.

(a) Average Cognitive and Metacognitive Activity			
Variables included	Beta	*t*	Sign.
Block 1 (Correct?)	0.751	9.20	0.0000
Pre Cognitive	0.252	3.07	0.0023
Post Metacognitive	0.223	2.73	0.0067

Variables excluded	Beta	*t*	Sign.
Post Cognitive	−0.172	−1.52	0.1292
Pre Metacognitive	−0.007	−0.06	0.9541

(b) Activity of Individual Regions			
Variables included	Beta	*t*	Sign.
Block 1 (Correct?)	0.738	9.17	0.0000
Pre Thalamus	0.317	3.93	0.0001
Post Mid Temp.	0.277	3.44	0.0007

Variables excluded	Beta	*t*	Sign.
Pre Occip./Temp.	0.738	−0.42	0.6721
Post Occip./Temp.	0.317	1.22	0.2246
Pre Mid Temp.	0.277	−1.56	0.1188
Post Thalamus	−0.041	1.29	0.1969
Pre Caudate	0.107	−1.70	0.0898
Post Caudate	−0.139	1.48	0.1408
Pre Frontpolar	0.113	0.67	0.5053
Post Frontopolar	−0.166	1.82	0.0689
Pre Frontal	0.137	−0.63	0.5309
Post Frontal	0.065	1.81	0.0715
Pre Angular Gyr.	0.153	0.05	0.9582
Post Angular Gyr.	−0.056	1.24	0.2148
Pre Parietal Lob.	0.151	−0.54	0.5897
Post Parietal Lob.	0.005	3.44	0.0896
Post Cent.l Gyr.	0.103	1.90	0.0585
Pre Cent.l Gyr.	−0.051	0.58	0.5635
Pre Medial Frontal	0.147	0.46	0.6446
Post Medial Frontal	0.157	1.21	0.2266
Pre SPFG.	0.055	0.59	0.5543
Post SPFG	0.041	−1.03	0.3018

Table 4b reports a stepwise regression analysis to localize which cognitive and metacognitive regions might be contributing most to this effect. We eliminated the two small cognitive regions (13 & 14 in Table 3) and averaged the left and right homologs of the metacognitive regions. This leaves 6 cognitive regions and 5 merged metacognitive regions. This analysis revealed a similar pattern of future success predicted by Pre activity in a cognitive region (the thalamus) and Post activity in a metacognitive region (the Middle Temporal Gyrus –MTG). Although these regions were the ones selected in the stepwise regression, there were a number of equivalent regions. Having entered the MTG, the Pre-response activity in any of the cognitive regions would have entered at *p*<.01. On the other hand, having entered the thalamus, only two of the metacognitive regions would have entered with a *p*<.01: Instead of the MTG, the Post response in the ANG could enter with a nearly equivalent *t* of 3.23 and it would then block entry of the MTG. Thus, all cognitive regions are predictive of future success to some degree and the MTG and ANG are nearly equally predictive.

To provide a converging test of the contribution of these regions we repeated the multiple regression analyses separately on the 36 cases for each exception type. The dependent variable was the number of correct solutions for that problem type on Blocks 2–8 and the five independent variables were correctness in Block 1, plus Pre and Post activity in the cognitive and metacognitive regions in Block 1. As before, the cognitive activity was the average of 6 of the cognitive regions used in the analysis of Table 3b. However, in this case the metacognitive activity was the average of the ANG and MTG only, as these were the only two metacognitive regions that were predictive in the combined-type analysis. We performed tests of whether the coefficients returned were reliable across the 9 exception types. There were significant effects of correctness in the Block 1 *t*(8) = 4.223 *p*<.005), of cognitive engagement before the response (*t*(8) = 3.23, *p*<.05), and of metacognitive engagement after the response (*t*(8) = 2.71, *p*<.05). Insignificant and negative coefficients were associated with cognitive engagement after the response (*t*(8) = −1.86 *p*>.1) and metacognitive engagement before the response (*t*(8) = −0.57, *p*>.5). This serves as a test of whether these effects generalize over problem types and do not reflect different activation patterns selecting different problem types that show different gains. Rather, the effects occur within each exception type.

Table 5 classifies the cases by whether the solution was correct on Block 1 or not, and whether the cognitive engagement and metacognitive engagements were above or below average. When participants correctly answered the first Exception Problem of a particular type, they are successful on about 6 or more of the remaining 7 instances. There is little room for contribution of engagement to the prediction. In contrast, if the participants failed to solve the same problem in Block 1, they averaged almost 2 more correct solutions when the relevant activity values were high. This is a 25% improvement in accuracy.

**Table 5:** Number Correct out of 7 on Blocks 2–8 as a function of Correctness on Block 1, Cognitive Engagement and Metacognitive Engagement.

	Wrong on Block 1	Wrong on Block 1	Correct on Block 1	Correct on Block 1
	Lo Post Meta	Hi Post Meta	Lo Post Meta	Hi Post Meta
Lo Pre Cognitive	3.69	4.49	6.04	5.91
Hi Pre Cognitive	4.83	5.44	6.10	6.40

**Figure 10:**
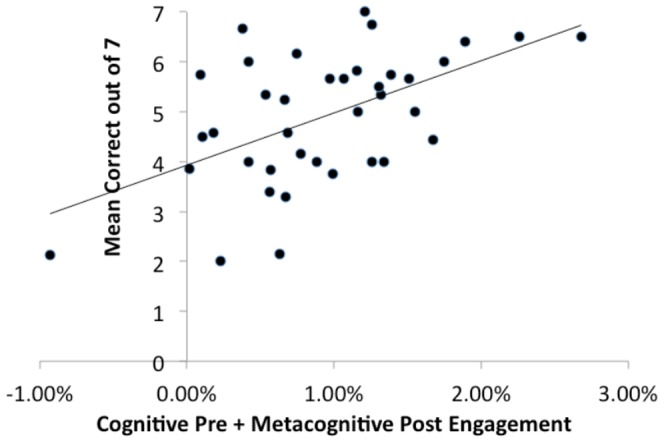
The relationship between participants' average Pre-cognitive engagement and Post-metacognitive engagement for incorrect Exception Problems in Block 1 and their success on those exception types in later blocks.

While the effects in Table 5 generalize across problem types, there remains the question of whether these Pre Cognitive and Post Metacognitive factors predict differences among participants, whether they predict differences within participants, or both. To address this question we performed two analyses focused on the problems answered incorrectly in first block (left half of Table 5). Both analyses looked at how the sum of Pre-Cognitive and Post-Metacognitive activity for these problems on Block 1 predicted number correctly solved on later blocks. To test whether there were also effects within participants we split these error Exceptions into cases where the summed activation was greater than that participant's average and the cases where it was below average. Only slightly more than half of the non-tie participants (15 out of 29) showed more correct solutions for the problems on which they showed more activation on Block 1. Thus, there is no evidence that activation differences within participants were predictive. To address the question of whether there were effects between participants, we looked at the correlation between the summed activation for a participant and the mean number that participant later solved correctly. As Figure 10 shows, there was a relatively strong relationship between the summed activation and future success across the participants (*r* = .54, *p*<.001). Thus, it seems that the relationship between activation and future learning is capturing differences between participants.

Cognitive engagement before a problem both predicts future success on that problem type and success on the problem itself. Ravizza et al. [12], who found that activity in the LIPFC predicted success on algebra problems, interpreted the greater activation as indicating that participants were engaged in a pattern of processing that would lead to a correct response. Cognitive engagement for incorrect responses may be associated with future success for similar reasons.

The two metacognitive regions (MTG and ANG) that predict future success are often associated with the language processing in the left hemisphere. The activations are bilateral in this task. A stepwise regression, given the choice between left and right regions, will choose the right, although they are nearly equivalent. Anderson et al. [18] reviewed the evidence that the ANG, particularly the right, is associated with reasoning about intentions. Also the MTG is involved in deductive reasoning [28], [29], reasoning about intentions [30], and arithmetic competence [31], [32].

### Patterns of Problem solving Engagement

**Figure 11:**
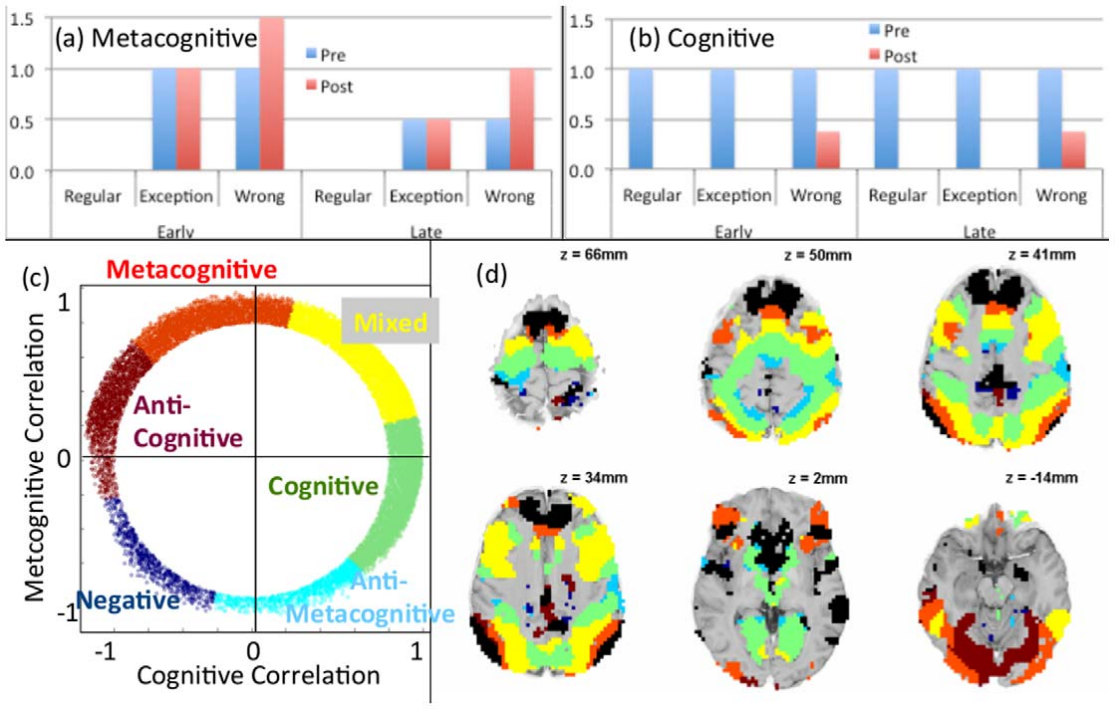
Correlation with metacognitive and cognitive patterns. (a) Metacognitive Pattern. (b) Cognitive Pattern. (c) Color coding of categories for 18,219 positively responding voxels with radius .8. (d) Brain distribution of voxels with radius >.8. Negatively responding voxels are in black and positively responding voxels use the color coding in (c). The value of z at each brain slice (shown in radiological convention: image left = participant's right) is at x = y = 0 in Talairach coordinates.

The exploratory analysis (Figure 7) revealed regions that showed relatively pure cognitive and metacognitive profiles. The predefined regions all showed a mixture of these profiles. Our earlier work (Figure 2) had revealed that there was actually a gradient of profiles across the brain. The current study offers added power to investigate the existence of such a gradient. To pursue this analysis requires specifying a complete Cognitive Pattern and Metacognitive Pattern for the current experiment. Figure 11 a&b provides such a specification in terms of the amount of engagement defined by crossing three variables:

1. Pre versus Post: activity before or after the response.
2. Correctly solved Regular Problems, correctly solved Exceptions, and incorrectly solved Exceptions, (not enough observations of incorrectly solved Regulars).
3. Early in practice (first two blocks) versus late in practice (last four blocks).

The exact values in the figure are somewhat arbitrary but they capture our qualitative assumptions: The Metacognitive Pattern only involves engagement for Exception Problems, which is reduced by 50% in the second half of the experiment. The Post engagement is equal to the Pre engagement for correct problems but increased by 50% if there is an error. The Cognitive Pattern is largely one of a simple engagement in the Pre Period and no engagement in the Post period. We assume that feedback on an error requires some cognitive engagement in the Post period. The exact amount (.375) was chosen to make the Cognitive and Metacognitive Patterns completely orthogonal.

We extracted measures of Pre and Post engagement for each voxel and calculated the correlation of these values with the Cognitive and Metacognitive Patterns in Figure 11a&b. Figure 11c shows the space of possible correlations. The radius of any point in this space reflects the combined correlation obtained with the two factors. The outer boundary in this scatter plot reflects the theoretical bound of 1 (the maximum combined correlation with two orthogonal factors). Figure 11c presents only those voxels with mean engagement greater than zero and radius greater than .8 (corresponding to an R^2^>.64 and a significance of p<0.01). Of the 40,429 positively responding voxels, 18,219 of these have radius greater than 0.8. Since we would only expect about 404 voxels by chance, it is apparent that these two patterns are accounting for real variance in the brain.

Using the convention in Anderson et al. [18], Figure 11c breaks these voxels into 6 color-coded 60-degree regions:

1. **Cognitive:** The 60-degree region from −45° to +15° where the correlation with the Cognitive Pattern is near 1.
2. **Mixed:** The 60-degree region from +15° to +75° where the correlations with the Cognitive and Metacognitive Pattern are about equal.
3. **Metacognitive.** The 60-degree region from +75° to +135° where the correlation with the Metacognitive Pattern is near 1.
4. **Anti-Cognitive.** The 60-degree region from +135° to +195° where the correlation with the Cognitive Pattern is near −1.
5. **Negative:** The 60-degree region from +195° to +255° where the correlation is about equally negative with the Cognitive and Metacognitive Patterns.
6. **Anti-Metacognitive.** The 60-degree region from +255° to +315° (or −45°) where the correlation with the Metacognitive Pattern is near −1.

Many of voxels in the Anti-Cognitive to Anti-Metacognitive range that had a radius greater than 0.8 were eliminated in Figure 11c because their mean engagement was negative.

Figure 11d shows the locations of all voxels with radius greater than 0.8, whether positively or negatively responding. The positively responding regions use the same color-coding as in Figure 11c, while the negatively responding voxels are in black. The similarity with the pattern in Figure 2 is quite striking. The greater power of this study (more participants, 12 rather than 8 points for the correlations) has resulted in somewhat more structure being revealed than our previous study. It is even more apparent that as one goes from the back to the front of the prefrontal cortex the activity changes from cognitive to metacognitive. In addition, in the posterior cortex there is a cognitive-to-metacognitive gradient going from medial to lateral regions. The negatively responding black regions often appear to be an extension of the metacognitive regions. These negatively responding voxels are in classic default network regions (e.g., [33], [34]) such as the medial prefrontal and posterior cingulate. The positively responding voxels include many of the regions already identified in the predefined or exploratory analyses. It is also striking just how much of the brain's activity correlates with the cognitive and metacognitive patterns. The challenging nature of the task means that it engages many processes.

## Discussion

This research replicates the earlier finding that these pyramid problems produce two neural patterns in a widely distributed set of regions. The cognitive pattern shows only shows high engagement while solving the problem and does not distinguish between Regular Problems and the different types of Exception Problems. While Exception Problems take longer and so evoke greater total activation, the amount of cognitive engagement per unit time does not differ among the problem types. In contrast, metacognitive regions tend to be engaged more after the problem is solved when feedback is presented. There are substantial differences in metacognitive activity among the problem types both before and after the response is given. These regions are found across the brain with the metacognitive regions more anterior in the prefrontal cortex and more lateral in the posterior cortex. Among the regions Anderson [11] and Dehaene et al. [8] associate with mathematical and algebraic problem solving, only the ANG shows the metacognitive pattern.

The current study also indicates that degree of metacognitive activation is not just a reflection of problem difficulty. In terms of accuracy, the three exception types were equally difficult and, in terms of latency, the Argument Exceptions were most difficult. However, Argument Exceptions, which require participants apply the same algorithm to more difficult numbers, produce the least metacognitive activity. The problems that produced greater metacognitive activation were problems that required changes to the algorithm, such as doing generate and test or not doing repeated addition at all (as in the mirror problems). These problems require participants to reflect on their algorithm for solving pyramid problems and to decide how to modify that procedure.

The current study found that metacognitive engagement for Exception Problems reduced with practice, although only for the Argument Exceptions did it go down to the level of activity associated with Regular Problems by the end of the 8 blocks. This is consistent with the assumption that these regions are related to developing mastery over these problems. As that mastery develops, the metacognitive engagement decreases.

Most significantly, engagement in the metacognitive regions after the response predicted future learning as did engagement in cognitive regions before the response. Given that cognitive engagement before the response also predicts success on the problem itself, it seems likely that it reflects the kind of problem solving that will lead to success. The metacognitive activation after the response presumably reflects successful processing of the feedback. Metacognitive activity before the response also significantly predicts successful learning, but its contribution is covered by the other two factors. On the other hand, there is no relationship between cognitive activity after the response and future learning.

With respect to the metacognitive regions, future success is only predicted by metacognitive activity in the MTG and the ANG. The Post-response activity in the other metacognitive regions does not have a significant relationship to future success. As we noted, left MTG and the ANG are associated with language processing and reading. While there is no verbal instruction in the feedback, making use of the feedback is a comprehension challenge. However, as we also noted, activity in the right homologs is at least as strongly predictive of future learning as the left activity. Although the language comprehension involvement of MTG and ANG tends to be associated with the left, the meta-analysis of Binder et al. [35] reports weaker activity in homologous right regions.

Anderson et al. [18] did not find a metacognitive region in the vicinity of the MTG but did find a similar pattern in the ANG. Thus, we should have more confidence that the ANG does play a critical role in solving these problems. The right ANG tends to be about two centimeters away from the center cited by Decety & Lamm [36] for the temporoparietal junction (ANG is a little higher and more posterior). Its coordinates do overlap with some studies reported in their meta-analysis. One theory-of-mind study found this region was more active when participants took another's perspective rather than their own [37]. This suggests that activation in the region might reflect trying to understand the experimenter's intentions in the definition of pyramid problems in order to correctly extend the definition to these Exception Problems. In addition, a right ANG region, basically identical to the predefined region, is activated by a mismatch between one's actions and the consequences of these actions [38]. Under these various interpretations it makes sense that the ANG should be engaged when reflecting on feedback for an Exception Problem.

More generally much of the area identified in Figure 11 seems is part of the fronto-parietal network that is engaged in many cognitive tasks [39]. There is considerable evidence that development of proficiency in mathematics is related to development of this network [40], [41]. With respect to kinds of problems studied in this paper, this developmental research involves relatively routine mathematical tasks that would activate the Cognitive network. It is an interesting question whether development of the cognitive-metacognitive gradient in Figure 11 is related to development of competence in more abstract mathematics.

The major focus of this research is on the metacognitive regions that become engaged with exception problems. The fact that they are more active for Algorithm Exceptions than Argument exceptions indicates that they are mainly driven by the need to reflect on changes to an established algorithm. Individuals who show greater levels of such activation are more successful in learning. The association of the predictive metacognitive regions with language comprehension suggests that language may be in some way the “carrier” of the reasoning required to master these novel concepts.
